# What Chemsex does to the brain - neural correlates (ERP) regarding decision making, impulsivity and hypersexuality

**DOI:** 10.1007/s00406-024-01856-2

**Published:** 2024-07-06

**Authors:** Johanna Schwarz, Marcus Gertzen, Andrea Rabenstein, Moritz Straßburger, Alana Horstmann, Oliver Pogarell, Tobias Rüther, Susanne Karch

**Affiliations:** 1 Department of Psychiatry and Psychotherapy, LMU University Hospital, LMU Munich, Germany; 2 Department of Psychiatry, Psychotherapy, and Psychosomatics, Medical Faculty, University of Augsburg, Augsburg, Germany

**Keywords:** Chemsex, Executive functions, Inhibition, Hypersexuality, Neurophysiological correlates

## Abstract

Chemsex describes the use of specific substances (methamphetamine, GHB/GBL, mephedrone, ketamine) which initiate or enhance sexual experiences and is mainly prevalent among men who have sex with men. Many Chemsex users experience somatic complications (for example sexually transmitted diseases) and sometimes adverse sociological, psychological, and neurological symptoms, such as depression, impulse control disorders or hypersexuality. Changes in impulsivity and deficits in executive functions have demonstrated to be associated with addiction and impulse control disorders as well as frontal brain dysfunction and behavioral control deficits. This study aims to explore the effects of neurophysiological correlates of inhibition and decision making in Chemsex users with an EEG paradigm using event-related potentials (N2, P3). 15 Chemsex users and 14 matched control subjects, all of them men who have sex with man, participated in an auditory Go/NoGo/Voluntary Selection EEG paradigm. In addition, clinical data (e.g. regarding depression), demographic information as well as measures of well-being and sexual behavior were collected. The results demonstrated that clinical symptoms, hypersexuality, and sexual risk behavior were more pronounced in Chemsex users compared to non-users. P3 amplitudes did not differ significantly between groups. However, the Chemsex users showed decreased electrophysiological N2 responses in fronto-central brain regions during decision-making, indicating compromised executive function and inhibitory control. The observed impairments may lead to increased risk behavior regarding drug abuse and hypersexuality. Understanding the neurobiological mechanisms can contribute to targeted interventions in order to mitigate the negative consequences of engaging in Chemsex and improve general well-being.

**Supplementary Information:**

The online version contains supplementary material available at 10.1007/s00406-024-01856-2.

## Introduction

Chemsex is an emerging global phenomenon of gaining importance [1]. It is defined as voluntary sex under the influence of specific substances (“chems”) and is predominantly practiced by men who have sex with men (MSM) just before or during sexual encounters [1].

David Stuart, one of the pioneers in defining Chemsex, specifically identified the primary substances associated to its practice: methamphetamine (“Crystal Meth”) and mephedrone, both synthetic stimulants, along with gammahydroxybutyrate/gamma-butyrolactone (GHB/GBL) [2]. Over time many authors have also included the dissociative anesthetic ketamine in the classification of substances associated with Chemsex [3]. Poly drug use is common [4] and Chemsex is typically practiced intermittently interspersed with episodes of abstinence [5]. The majority of Chemsex users reports that the substances aid in facilitating, enhancing, prolonging and sustaining their sexual experience [4]. However, regular practice of Chemsex often leads to negative consequences. These consist of physiological effects such as contracting multiple sexually transmitted diseases (STD’s) [6], interpersonal and social consequences like experiencing abuse during sexual sessions, loss of control, and difficulties managing work or daily life [7]. Additionally, psychological effects including anxiety, depression and psychosis [4, 7, 8] as well as neurological manifestations such as convulsions [4] have been observed. Despite not recognizing their substance use as an addiction, Chemsex users exhibit similarities to individuals with addictive disorders including impaired control over consumption, physiological tolerance development, and prioritization of chemsex practices over other life aspects [9].

Up to 22% of MSM living with HIV have displayed a higher degree of compulsive sexual behavior and diminished impulse control, leading to an intense preoccupation with sexual desires and inability to restrain them [10]. Consequently, they engage in more frequent and unsafe sexual encounters, commonly referred to as “hypersexuality”. It is characterized by an overwhelming and uncontrollable fixation on sexual fantasies, urges, and behaviors, resulting in loss of control, distress, and other negative consequences [10–12]. For instance, those affected frequently tend to engage in risky sexual behavior to cope with stress or negative emotions. This can lead to conflicts in other areas of their lives and poses a potential harm to themselves or others [11, 12]. Despite attempts to control or decrease these behaviors, individuals with hypersexuality frequently find themselves repeating recurrent patterns, returning to previous behaviors [11, 12]. Consequently, hypersexuality is linked to difficulties in controlling impulses.

Impulsivity seems to play a major role in addictive behavior. It is a personality trait which exists on a continuum of varying degrees. It can be useful and necessary for the rapid execution of tasks. However, very high impulsivity involves a predisposition to respond to internal or external stimuli with a swift and unplanned reaction without considering the negative consequences for oneself or the environment [13]. Impulsive behavior often appears situationally inappropriate and uncontrolled to outsiders. The aspects of impulsivity include quick decisions, intolerance to reward delay, lack of perseverance, and inflexibility [13]. Dawe et al. (2004) found that impulsive behavior is often related to drug abuse with substance misusers scoring higher on impulsivity measures such as novelty-seeking, sensation-seeking, and behavioral control [14]. Additionally, children with measured impulsiveness appeared to be at higher risk of developing substance use disorders as adults [14].

Impulsivity involves neurobiological mechanisms, with particular emphasis on the prefrontal cortex, as its impairment has been linked to the inability to inhibit compulsive behavior [14]. Deficits in executive functions [15–18] and behavioral control [19, 20] are associated with changes in impulsivity. Disinhibition resulting from impulsivity can lead to behavior that is primarily determined by previously conditioned responses, which may be inappropriate for the current circumstances [21]. The impulsivity-associated neuropsychological dysfunctions of the frontal lobe often lead to the inability to perform complex psychomotor tasks and other frontal lobe-related skills, such as problem solving [22]. Evidence points not only to the involvement of frontal brain structures, but also fronto-striatal brain regions. These include the dorsolateral prefrontal cortex, anterior cingulate cortex (ACC), basal ganglia, thalamus, ventromedial temporal cortex, and medial parietal/posterior cingulate [22–28].

One of the factors contributing to prefrontal impairment is drug abuse. Long-term exposure to drugs can directly negatively affect frontal cortex regions involved in inhibitory response control, such as the fronto-striatal system or neural dopaminergic projections and pathways [21]. Studies have demonstrated that drug addiction is associated with impaired brain function and neural correlates, including lower activity in prefrontal cortical regions [29, 30]. This is a particularly interesting fact, as substances increase impulsivity and, conversely, deficits in this area increase drug use. Additionally, substance abuse has been shown to cause lasting damage to neural structures and circuitry, that also lead to impulse control and compulsive disorders [31]. Individuals who suffered from alcoholism exhibited metabolic abnormalities, such as decreased glucose utilization, and reduced blood perfusion, particularly in frontal brain regions [17, 22, 23, 25].

In the pursuit of deeper insights into the underlying neural mechanisms of cognitive deficits regarding impulsivity and addiction, scientists have employed event-related potential (ERP) techniques that are measured using electroencephalography (EEG). ERPs are timed measurements of electrical brain activity that represent a specific phase of cortical processing [32]. These approaches enable the investigation of brain activity patterns associated with drug addiction. Executive functions, for example decision making, impulsivity and behavioral inhibition, are often addressed using so called *Go/NoGo* paradigms: In these paradigms, participants are instructed to rapidly respond to *Go* trials while withholding behavioral responses on *NoGo* trials. Two components of the ERPs measured in such tasks are the N2 negativity and the P3 positivity, which occur approximately 200ms and 300ms after the stimulus, respectively [32]. The N2 and P3 components emanate from the ACC and are employed in such *Go/NoGo* tasks to measure behavioral suppression and cognitive control [33]. Given the established association between deficits in these cognitive domains and impulsivity, ERPs are consequently employed to quantify impulsivity. Studies have consistently revealed frontocentral activity (N2, P3) linked to behavioral inhibition [34–36]. Specifically, increased N2 and P3 responses are observed when a response is withheld (*NoGo* trials) within a series of *Go* trials [36–39]. The ACC has also been shown to be active during voluntary-decision making, particularly in scenarios involving conflicting response trials with simultaneous incompatible response tendencies [38]. Modified ERPs have been observed in individuals with addiction disorders. Blunted N2 amplitudes, particularly during the *NoGo* condition, have been found in people with addictions to heroin [40], nicotine [41] and even internet [42, 43]. Decreased P3 amplitudes have been detected in subjects with addictions to alcohol [44], heroin [45], GHB [46] and ecstasy [47]. Additionally, a study focusing on cocaine abusers revealed hypoactivity of higher-level executive motor control attributed to the prefrontal cortex and an impairment of the ACC [48].

In the present study, we examined neurobiological correlates (ERPs) of Chemsex users and a control group by applying a *Go/NoGo* EEG-paradigm with an additional *Voluntary selection* condition to assess decision-making and impulsivity. Additionally, we conducted clinical data measuring depression and well-being, hypersexuality and sexual risk behavior.

## Methods

### Subjects

43 adult MSM (23 Chemsex users and 20 control subjects) were examined in the EEG study. The Chemsex users included were mainly acquired through our Chemsex outpatient clinic of the LMU University Hospital, LMU Munich. The control group was reached largely by distributing flyers in queer bars and restaurants as well as in medical practices specializing in treating and preventing HIV by prescribing Pre-Exposure Prophylaxis (“PrEP”). Inclusion criteria for both groups were age between 18 and 60 and identification as MSM. A criterion exclusively for the Chemsex group was the use of one or more main substances (methamphetamine, ketamine, GHB/GBL, or mephedrone) in a sexual context at least once within the past six months. The control subjects consisted exclusively of individuals who had never used any of the four main substances in a sexual context. All participants received instruction to abstain from drug consumption (except for nicotine) and sexual encounters in the 24 h prior to the experiment. Subjects with serious medical conditions, acute intoxication, suicidal or psychotic states were excluded from the study. The subjects of both groups were matched with each other for age and level of education.

A total of 14 participants (8 Chemsex users and 6 control subjects) were excluded from the data analysis: 2 participants exhibited auditory impairment and were not able to distinguish the different auditory stimuli, 1 subject displayed artefacts due to consistent eye movements, and 11 subjects demonstrated incomplete comprehension of the task and its conditions (by pressing the button less than 10 times or exceeding 90 times after the *Voluntary selection* stimulus). 29 subjects were included in the analysis: 15 Chemsex users (aged between 36 and 58 years; mean age 44.67 ± 8.97) and 14 control subjects (aged between 21 and 57 years; mean age 39.71 ± 11.03). The two groups did not differ significantly in terms of age [T = -1.33; *p* = 0.194] and education level (60% of Chemsex users and 71,4% of the control subjects had a university degree) [*p* = 0.518] (Table 1).

**Table 1:** Demographic characteristics of chemsex and control group

	Chemsex group	Control group	*p*
Age (M ± SD)	44.67 ± 8.97	39.71 ± 11.03	0.194
Age (Range)	36–58	21–57	-
Education level (% of participants with university degree)	60	71.4	0.518
Psychiatric treatment in the past (%)	33.3	42.9	0.597
Current permanent relationship (%)	66.7	71.4	0.782
Number of sexual partners in the last 6 months (M ± SD)	15.60 ± 9.575	11.00 ± 15.072	0.337
Current or past STD (%)	93.3	64.3	0.054
Consumption of			
Alcohol (%)	93.3	100.0	0.326
Nicotine (%)	46.7	42.9	0.837
Cannabis (%)	33.3	71.4	0.040
Methamphetamine (%)	93.3	7.1	< 0.001
GHB/GBL (%)	80.0	28.6	0.005
Mephedrone (%)	46.7	0.0	0.003
Ketamine (%)	46.7	14.3	0.060
Cocaine (%)	73.3	28.6	0.016
MDMA (%)	53.3	35.7	0.340
Amphetamine (%)	33.3	21.4	0.474
Poppers (%)	100.0	50.0	0.002

M = mean value, SD = standard deviation, % = percentage, *p* = level of significance, Consumption of = the participants stated to have used this substance at least once in their life

Written informed consent was obtained from each participant after study procedures and privacy measures were fully explained. The study was previously approved by the ethics committee (No. 18–833) of the LMU University Hospital Munich and conducted in accordance with the Declaration of Helsinki. Each volunteer of the control group was paid 50€ for participating in the study.

### EEG procedure and paradigm

**Fig. 1:**
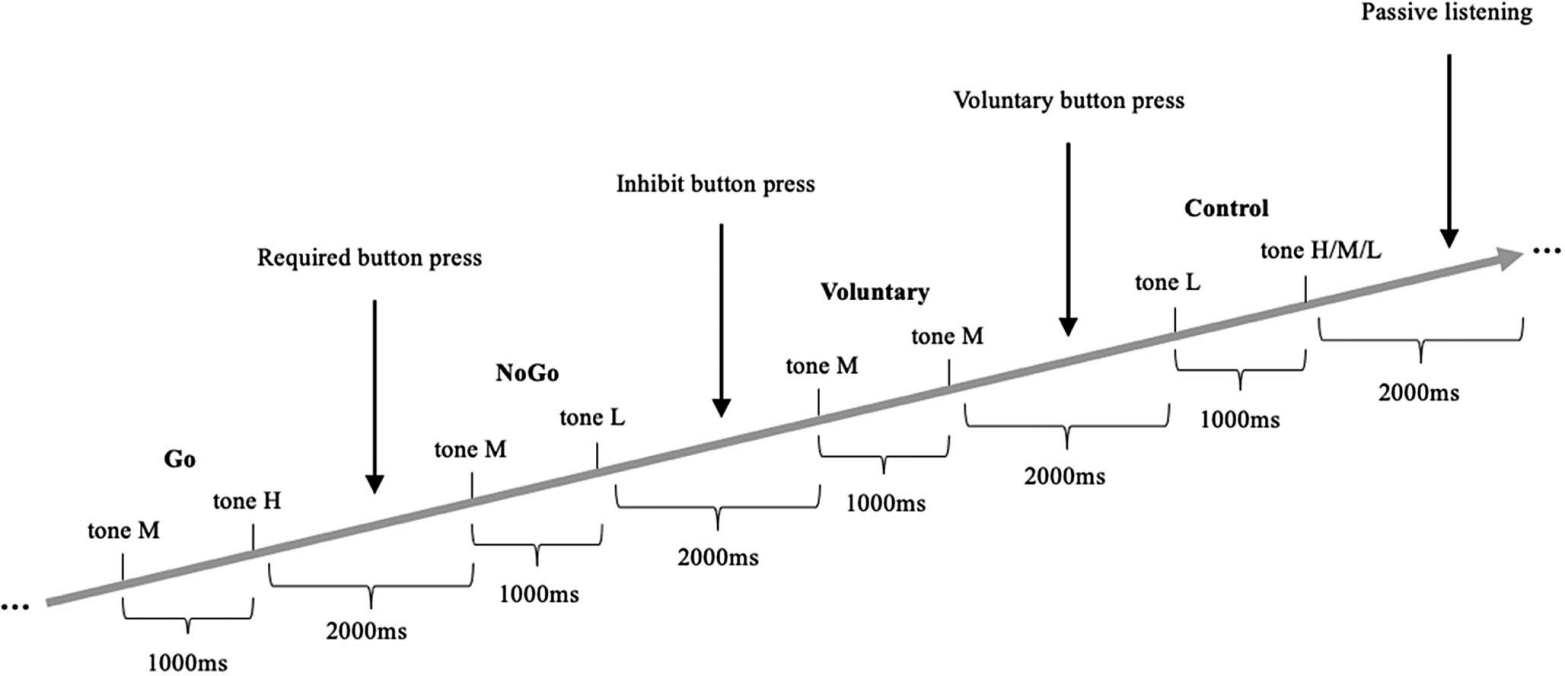
Exemplary sequence of presented tone pairs. ms = milliseconds

Subjects performed a *Go/NoGo/Voluntary* EEG paradigm involving auditory stimuli, which are suitable for investigating cognitive processes such as response inhibition and attention. Auditory stimuli offer advantages in mitigating potential confounds associated with eye movements and attention shifts often encountered with visual stimuli [49]. We deliberately abstained from employing sexual or drug-related stimuli to preclude potential biases in attention, cognitive processing, and response tendencies between the Chemsex and control group, which could arise from different attitudes towards such content. The EEG paradigm consisted of sinusoidal tones (pressure level of 100 dB) with three different pitches (800, 1000 and 1300 Hz), delivered binaurally via headphones. The tones were 50ms long and presented in pairs of intervals of 1000ms. The subsequent trial was presented 2000ms after the second tone. The tone with the middle frequency (1000 Hz) served as a cue indicating the requirement for a reaction. Participants kept their dominant index finger on the button of the response box. Notably, three out of the 15 Chemsex patients and one out of the 14 control subjects were left-handed. The rest stated to be right-handed. They were instructed to press the button with their dominant hand as quickly as possible when the middle frequency tone was immediately followed by the high frequency tone (1300 Hz; *Go* condition), all while minimizing errors. In the *NoGo* condition, the cue tone was followed by a low frequency tone (800 Hz), and subjects were instructed to inhibit their responses by not pressing the button. In the *Voluntary Selection* condition, the cue was followed by a tone with the same frequency (1000 Hz) and participants were allowed to freely decide whether to press the response button (*Selection +*) or not (*Selection -*). Subjects were asked to independently choose whether they wanted to respond or not during the *Voluntary Selection* task, with the aim of having approximately equal frequency of button presses and non-presses in random order. In addition, subjects were instructed not to count how often they pressed the button and not to alternate between button press and not press. Subjects who pressed the button after less than 10% or more than 90% of the *Voluntary Selection’s* tones were excluded from the study, because it could not be guaranteed that they had understood the instructions. Finally, the paradigm contained two passive listening tasks where participants were not required to respond regardless of the second tone’s frequency. Those tasks always started with the low-frequency tone (800 Hz; *Control* condition). All conditions were presented in pseudo-randomized order, with the *Go* condition being presented 200 times and all other conditions being presented 100 times each. An overview of all tone qualities and number of trials is presented in Table 2, an exemplary sequence of different tone pairs is presented in Fig. 1. Prior to recording the EEG, all subjects received a practice block of at least 10 min to familiarize themselves with the different response rules and ensure complete understanding of the instructions. Also, the practice block should ensure normal hearing of all participants. The auditory stimuli were generated using the Presentation software package (Neurobehavioral Systems, http://neurobs.com) on a computer placed outside the room where subjects were seated comfortably in chairs while the EEG was recorded.

**Table 2:** Frequency of the tone qualities used in the experiment, and number of trials with the corresponding experimental condition

Tone qualities		Conditions	
M	middle frequency (1000 Hz)	Go	M + H (200 trials)
L	low frequency (800 Hz)	NoGo	M + L (100 trials)
H	high frequency (1300 Hz)	Voluntary	M + M (100 trials)
		Control	L + H/M/L (100 trials)

Hz = Hertz

### Recording of behavioral data and analysis

Behavioral data were gathered using the Presentation software package (Neurobehavioral Systems, http://neurobs.com). Reaction times (RTs), errors of omission (during *Go* condition) and commission (during *NoGo* condition) were recorded. In the *Go* condition, response delays exceeding 1500ms after stimulus presentation were regarded as errors. Responses faster than 50ms were considered anticipatory responses and counted as errors as well. In the *Voluntary Selection* condition, button presses occurring within the 0-1500ms after stimulus presentation were categorized as *Selection +*, while trials without behavioral response within the first 1500ms were classified as *Selection -*. Mean RTs for each condition (*Go, Voluntary Selection +*) and for each subject were calculated separately. To compare response times and error rates, a repeated-measurement ANOVA was conducted, utilizing Chemsex group and control group as between-factors.

### EEG acquisition and data analysis

Event-related potentials were recorded using a setup with 27 electrodes, all referenced to Cz. The electrodes placement on the scalp followed the international 10–20 system, using an electrode cap set (Easycap, Germany). A ground electrode was integrated in the cap. The specific electrode positions included Fp1, Fp2, F3, F4, C3, C4, P3, P4, O1, O2, F7, F8, T3, T4, T5, T6, Fz, Cz, Pz, Fc1, Fc2, Fc5, Fc6, Cp5, Cp6, P9, P10, with additional electrodes at T1, T2, A1 and A2. Eye movements were recorded using a channel beneath the left eye (EOG). The EEG signals were continuously recorded and digitalized at 5000 Hz without any filter during acquisition. Electrode impedances were typically maintained below 5 kΩ. An amplifier designed for inside scanner recordings (Brain Products, Munich) was used for EEG acquisition. During the task, participants were instructed to remain calm and keep their eyes closed. Recording took place in a sound-attenuated and electrically shielded room.

Data analysis was performed using the Brain Vision Analyzer Software (Brain Products, Munich). Channels A1 and A2 were excluded from analysis due to continuous artefacts in most data sets. The data were re-referenced to a common average reference and run through a Zero phase shift Butterworth filter using a 1 Hz-low cutoff, a 100 Hz-high cutoff, and a 50 Hz-notch filter. The recordings were segmented into 2750ms epochs, time-locked 200ms before first stimulus of each pair of tones, separately for different conditions (*Go, NoGo, Voluntary Selection, Control*).

The sampling epoch started 1000ms before the presentation of the second tone that indicated which task was to be performed. An amplitude criterion of 70 mV was used for artefact rejection, including all channels at any time point during the averaging period. For baseline-correction, the 200ms interval before the presentation of the second stimulus of each pair of tones was used. ERP waveforms were averaged separately for each task condition. Trials with incorrect responses (button press after *NoGo* or *Control* tasks; no response after *Go* tasks) were excluded prior to averaging. The N1, N2 and P3 ERPs were examined at the midline fronto-centro-parietal scalp electrodes (Fz, Cz, Pz). The N1 was defined as the relative minimum of the ERP at electrode in the search window of 50.0–150.0ms. The N2 was defined as the largest relative minimum of the ERP in the search window of 150–250ms. The P3 was defined as the largest relative maximum of the ERP 250–650ms after the presentation of the respective task.

### Clinical data and questionnaires

Further data was collected, including the Beck Depression Inventory (BDI) a self-rated questionnaire [50] and the Montgomery-Åsberg Depression Rating Scale (MADRS) as an expert-rated questionnaire [51] to assess the potential severity of depression. In addition, participants filled out a survey concerning their demographic data, sexuality and Chemsex behavior (only applicable to the Chemsex group), as well as the Hypersexual Behavior Inventory (HBI), a psychological, self-rating assessment tool to measure the severity of hypersexual behavior [11, 52]. All participants rated their physical and mental well-being, as well as their sexual risk behavior on a Likert-scale from 1 to 10 (higher scores indicating better well-being and safer sexual behavior, respectively). To evaluate substance use, participants were queried about their use of 23 different psychotropic substances, specifically whether they had consumed each substance at least once.

### Statistics

Repeated measurement ANOVAs were calculated for the maximum ERP-amplitude in each interval (N1, N2, P3) with the two repeated-measurement factors task (*Go +, NoGo -, Voluntary Selection +/- and Control*) and electrode position (Fz, Cz, Pz) and one between subject factor group (Chemsex users: “CHSX”, and control subjects: “Control”). In case of a significant Mauchly-test, the Greenhouse-Geisser correction was applied. Additionally, post hoc t-tests were performed with Bonferroni correction for multiple tests: *p*-values smaller than 0.05 were considered significant, *p*-values smaller than 0.1 were considered trend. The relationship between reaction times, amplitudes, demographic parameters, MADRS, BDI and HBI, were calculated using the Pearson correlation coefficient for interval-scaled variables and the Spearman correlation coefficient in case of at least one nominal-scaled variable. Furthermore, we calculated t-tests or χ2-tests to compare specific questionnaire parameters between Chemsex users and the control group.

## Results

### Questionnaires: sexuality and well-being

**Fig. 2:**
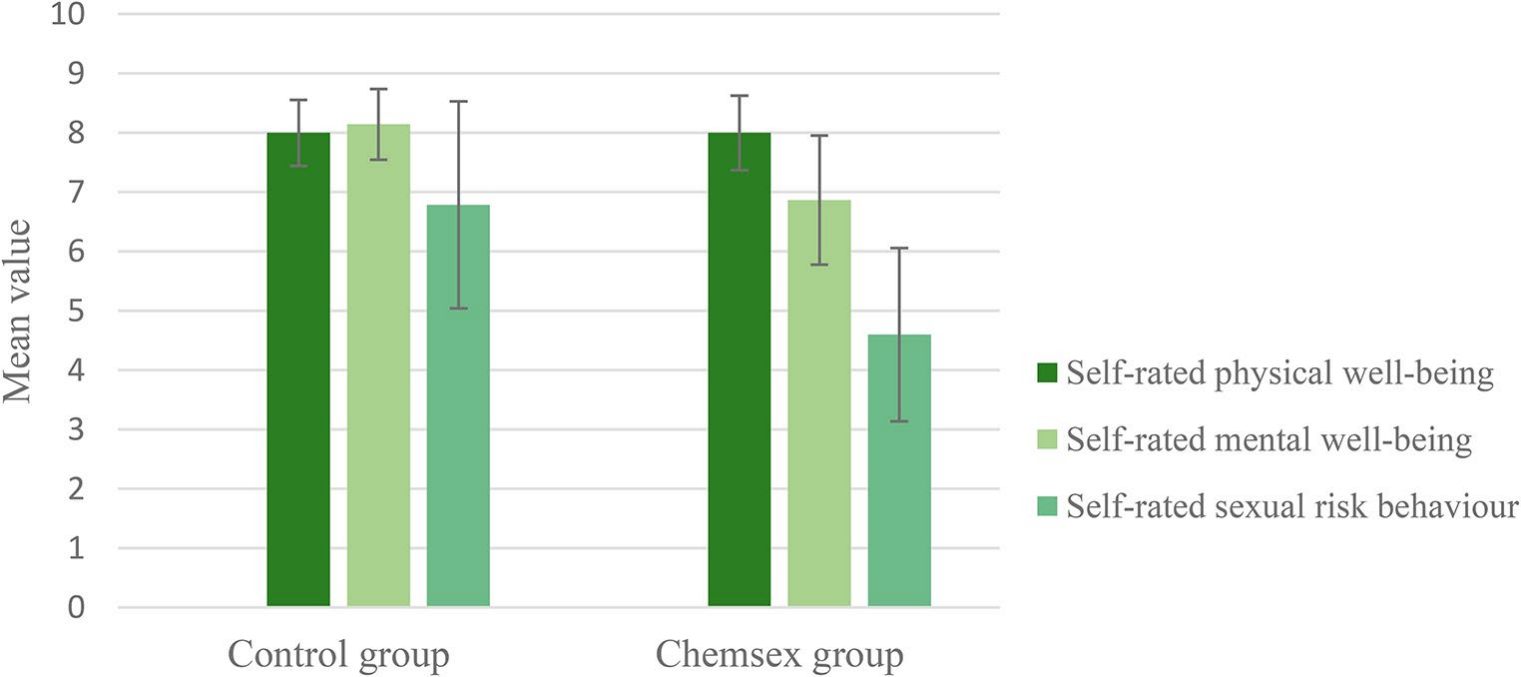
Mean value per group for self-rated physical and mental well-being as well as sexual risk behavior

Apart from one individual in the control group who identified as a bisexual male, the entire study population consisted of self-identified homosexual males. 71.4% of the control and 66.7% of the Chemsex group stated to be in a permanent relationship [χ2 = 0,077; *p* = 0.782]. The mean duration of partnerships at the time of the survey was 11.00 ± 9.00 years for the control and 7.65 ± 11.884 years for the Chemsex group [T = 0.660; *p* = 0.260]. The Chemsex users reported slightly more sexual partners within the last six months (15.60 ± 9.575) compared to the control group (11.00 ± 15.072). The difference was not significant [T = -0.978; *p* = 0.337]. 64.3% of the control group and 93.3% of the Chemsex group stated to have or have had a STD [χ2 = 3.724; *p* = 0.054]. 28.6% of the control group and 46.7% of the Chemsex users reported an infection with the Human Immunodeficiency Virus (HIV) [χ2 = 1.007; *p* = 0.316]. There was no significant difference regarding the physical well-being of each group [CHSX: M = 8.00 ± 1.134; Control: M = 8.00 ± 0.961; T = 0.00; *p* = 1.00]. However, mental well-being differed significantly between the groups [CHSX: M = 6.87 ± 1.959; Control: M = 8.14 ± 1.027; T = 2.173; *p* = 0.039], as did sexual risk behavior [CHSX: M = 4.60 ± 2.640; Control: M = 6.79 ± 3.017; T = 2.080; *p* = 0.047] (Fig. 2).

**Fig. 3:**
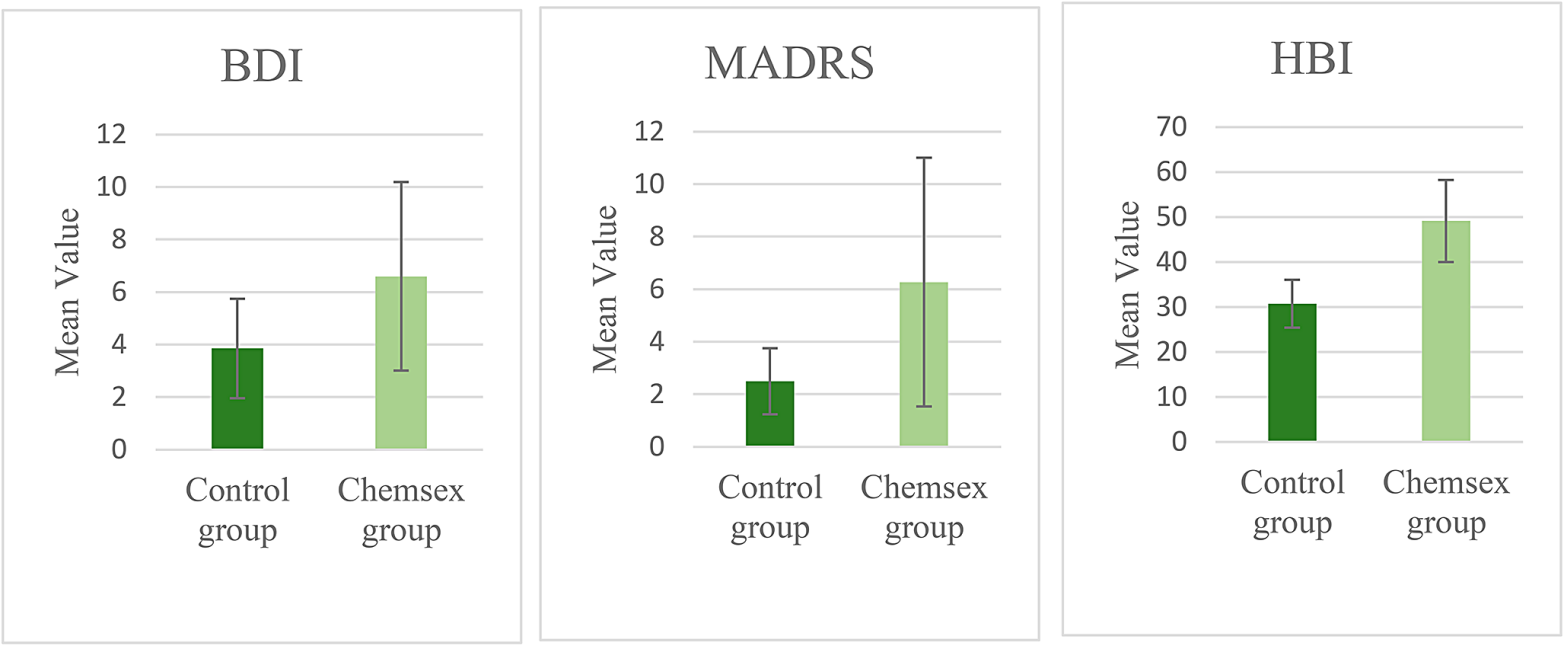
Comparison of psychometric data between Chemsex users and controls. BDI = Beck Depression Inventory, MADRS = Montgomery-Åsberg Depression Rating Scale, HBI = Hypersexual Behavior Inventory

The BDI score and the MADRS were slightly higher in the Chemsex group compared to the control group (Fig. 3); however, differences were not significant [BDI: CHSX: M = 6.60 ± 6.490; Control: M = 3.86 ± 3.278; T = -1.420; *p* = 0.167; MADRS: CHSX: M = 6.27 ± 8.548; Control: M = 2.50 ± 2.175; T = -1.650; *p* = 0.118]. In terms of hypersexuality, the Chemsex users exhibited significantly higher results in the HBI than the control group [CHSX: M = 49.13 ± 16.475; Control: M = 30.71 ± 9.227; T = -3.746; *p* = 0.001] (Fig. 3). Additionally, 6 individuals from the control and 5 individuals from the Chemsex group reported having undergone psychiatric or psychotherapeutic therapy in the past. Regarding psychotropic medications only participants within the Chemsex group reported any use of medication: one person stated to take Olanzapine, two people reported taking Buproprion, one participant reported taking Escitalopram and another Citalopram and Mirtazapine.

### Questionnaires: Chemsex behavior and substance use

The questions pertaining to Chemsex were exclusively answered by the Chemsex users. The participants of this group had an age of 39.81 ± 7.607 when they first engaged in a Chemsex session. On average, the group had their first Chemsex session averagely 4.85 ± 6.309 years prior to participating in the study. The participants reported an average of 11.07 ± 10.512 sessions within the past six months and a mean duration of 30.60 ± 46.760 days since their last session. On a scale from 1 (minimum) to 10 (maximum) they rated their feeling of disinhibition during a session with a mean score of 9.07 ± 0.594), their feeling of suffering regarding their Chemsex behavior with a mean score of 5.60 ± 3.291, and their perceived Chemsex addiction with a mean score of 5.80 ± 2.624. Notably, 9 out of the 15 patients (60%) expressed a desire to refrain from Chemsex.

Regarding substance use significant differences between the two groups were observed in percentage of users of methamphetamine [CHSX: 93.3%; Control: 7.1%; χ2 = 21.544; *p* < 0.001], GHB/GBL [CHSX: 80.0%; Control: 28.6%; χ2 = 7.744; *p* = 0.005], mephedrone [CHSX: 46.7%; Control: 0.0%; χ2 = 8.612; *p* = 0.003], cocaine [CHSX: 73.3%; Control: 28.6%; χ2 = 5.811; *p* = 0.016], poppers [CHSX: 100.0%; Control: 50%; χ2 = 9.886; *p* = 0.002]. The consumption of ketamine reached trend level [CHSX: 46.7%; Control: 14.3%; χ2 = 3.548; *p* = 0.060]. No significant differences were detected between the two groups in relation to other substances, such as alcohol [CHSX: 93.3%; Control: 100.0%; χ2 = 0.967; *p* = 0.326], nicotine [CHSX: 46.7%; Control: 42.9%; χ2 = 0.042; *p* = 0.837], MDMA [CHSX: 53.3%; Control: 35.7%; χ2 = 0.090; *p* = 0.340], and amphetamine [CHSX: 33.3%; Control: 21.4%; χ2 = 0.514; *p* = 0.474]. The control group showed a significantly higher number of cannabis users [CHSX: 33.3%; Control: 71.4%; χ2 = 4.209; *p* = 0.040].

### EEG paradigm: behavioral results

Behavioral data are shown in Table 3.

**Table 3:** Behavioral data of chemsex and control group

	Chemsex group		Control group	
	M	SD	M	SD
Reaction times (ms)				
Go	429.1	74.1	398.5	65.1
Selection +	671.2	100.8	628.3	110.1
Percentage of responses (%)				
Go	97.1	3.0	97.4	2.5
Selection +	62.0	12.0	60.3	18.4
Percentage of mistakes (%)				
Go	2.6	2.6	2.6	2.5
NoGo	1.7	2.0	1.1	1.5

M = mean value, SD = standard deviation, ms = milliseconds, % = percentage, mistake = missing button press despite Go cue or button press despite NoGo cue

In *Voluntary Selection +* trials, the group mean RTs were found to be significantly longer than in *Go* trials [F(1;27) = 207.554; *p* < 0.001]. Although the Chemsex users demonstrated slightly slower reaction times compared to the control group, this difference was not statistically significant [F(1;27) = 1.617; *p* = 0.214]. In addition, the interaction effect (condition × group) was not statistically significant [F(1;27) = 0.140; *p* = 0.711]. We did not find any differences regarding the percentage of reactions during the *Voluntary Selection* task [group effect: F(1;27) = 0.057; *p* = 0.813; interaction effect: F(1;27) = 0.122; *p* = 0.730].

The percentage of incorrect responses after the mandatory cues was significantly higher after the *Go* stimulus compared to the *NoGo* stimulus [F(1;27) = 5.939; *p* = 0.022). Both, the differences between patients and healthy subjects [F(1;27) = 0.235; *p* = 0.632] as well as the interaction effect [F(1;27) = 0.526; *p* = 0.475] were not significant.

### EEG paradigm: ERP results

**Fig. 4:**
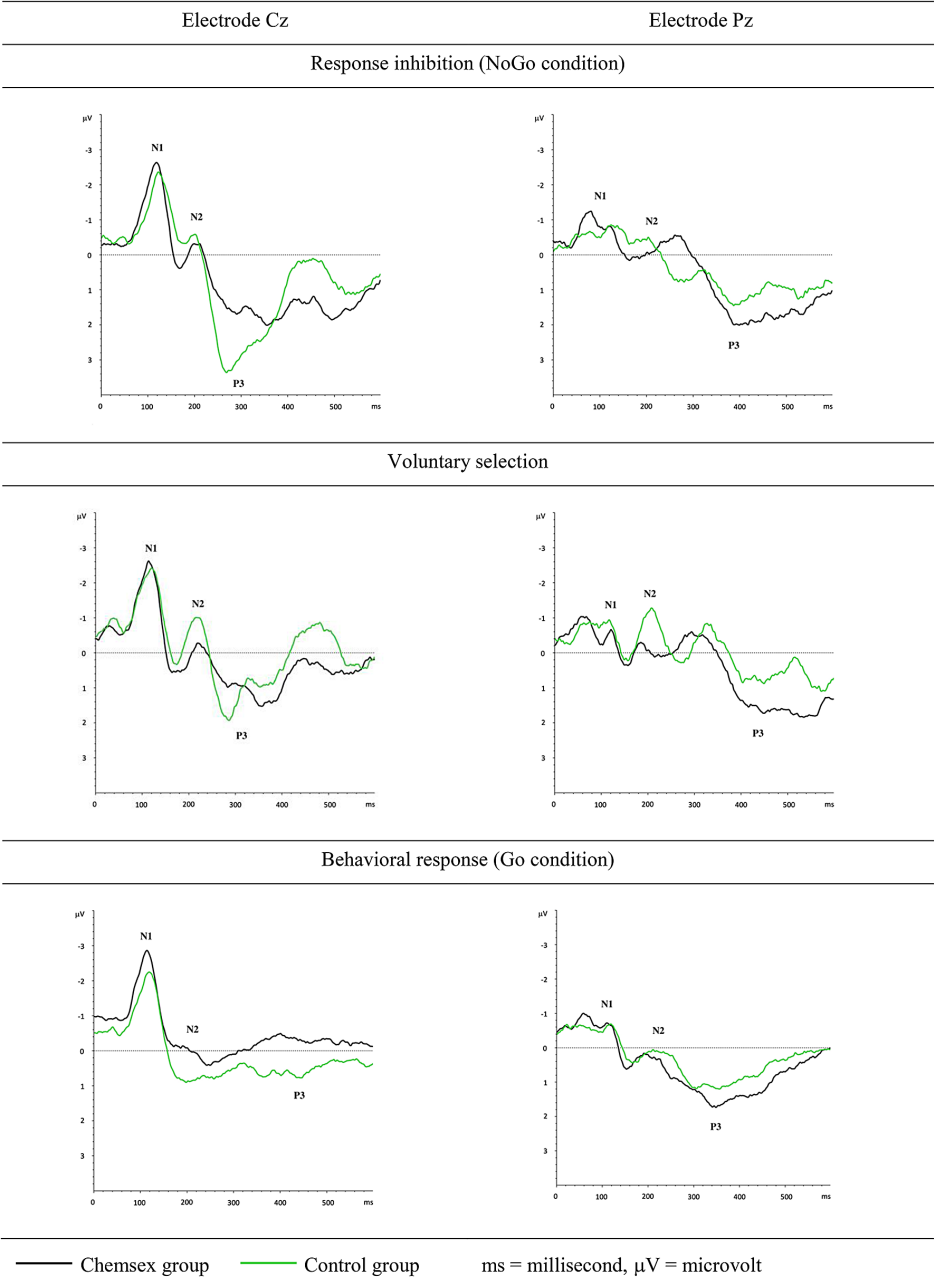
Inhibition-associated ERP waveforms of chemsex users and control subjects at fronto-central and parietal sites

A repeated measurement ANOVA was calculated with the two repeated-measurement factors task (*Go +, NoGo -, Voluntary Selection +/- and Control*) and electrode position (Fz, Cz, Pz) and one between subject factor group (Chemsex users and control subjects). Results are shown in Fig. 4.

The N2-amplitudes differed significantly between conditions [F(2,725;108) = 15.219, *p* < 0.001]. Post hoc tests indicated that the N2 was less prominent in the *Go* condition compared to the *NoGo* (*p* = 0.006), *Voluntary Selection +* (*p* < 0.001) and *Voluntary Selection -* (*p* < 0.001) conditions. Similarly, the N2 amplitude was less prominent in the *Control* condition compared to the *NoGo* (*p* < 0.001), *Voluntary selection +* (*p* < 0.001) and *Voluntary Selection -* (*p* < 0.001) conditions. Regarding electrode position, the differences in N2-amplitudes reached trend level [F(2;54) = 3.160, *p* = 0.50): the N2 amplitudes were comparable in Fz and Cz (*p* = 1.000); small differences were demonstrated between Pz and Fz (*p* = 0.123) and Pz and Cz (*p* = 0.112). The interaction effects of condition × electrode position [F(8:216) = 3.725; *p* < 0.001], condition × group [F(2,725;108) = 3.531, *p* = 0.022] and electrode position × condition × group [F(8;216) = 2.235; *p* = 0.026] were significant. There was no significant interaction effect between electrode position and group [F(2;54) = 0.608, *p* = 0.548] and no significant group effect between Chemsex users and control group [F(1;27) = 1.022, *p* = 0.321]. However, there were observable differences between groups, for example shallower N2 amplitudes (Cz) of Chemsex users in the *NoGo* and *Voluntary Selection* condition. T-tests were calculated to assess the differences between groups for each electrode separately. The difference of the N2 Cz amplitude between the Chemsex and the control group reached trend level for the *NoGo* condition [CHSX: M = -1.674; Control: M = -2.423; T = -1.358, p (one-sided) = 0.093] and statistical significance for the *Voluntary Selection +/-* [CHSX: M = -1.959; Control: M = -1.046; T = -1.806; p (one-sided) = 0.041].

Regarding P3-amplitudes the ANOVA presented significant main effects for condition [F(3,042;108) = 40.690; *p* < 0.001] and electrode positions [F(2;54) = 4.120; *p* = 0.022], as well as significant interactions between condition and electrode position [F(5,244;216) = 6.692; *p* < 0.001], and electrode position × group [F(2;54) = 3.500; *p* = 0.037]. Post hoc tests revealed that the P3 amplitudes were larger in *NoGo* and *Voluntary Selection +/-* conditions compared to *Go* and *Control* conditions (*p* < 0.001). The P3 amplitudes did not differ significantly between Fz and Cz (*p* = 0.152) and between Cz and Pz (*p* = 1.000). The difference between the electrodes Fz and Pz reached trend level (*p* = 0.063), with increased amplitudes at Pz compared to Fz. Although the P3 amplitudes in Fz and Cz were smaller and the P3 amplitudes in Pz appeared to be higher in the Chemsex group compared to the control group, the main effect of group was not significant [F(1;27) = 0.000; *p* = 0.991]. Furthermore, the interaction effects were not statistically significant [condition × group: F(3,042;108) = 0.355; *p* = 0.788; condition × electrode position × group: F(5,244;216) = 0.647; *p* = 0.671]. T-tests revealed a significant difference at Fz between the two groups regarding the *Go* condition [CHSX: M = 0.477; Control: M = 0.916; T = 1.973; p (one sided) = 0.029]; differences between groups reached trend level regarding the *NoGo* condition [CHSX: M = 2.537; Control: M = 3.159; T = 1.410; p (one sided) = 0.085] and showed no significance regarding the *Voluntary Selection* condition [CHSX: M = 2.442; Control: M = 2.258; T = -0.495; p (one sided) = 0.312]. Differences between groups were not significant at Pz for the *Go* condition [CHSX: M = 2.382; Control: M = 1.933; T = -1.139; *p* = 0.132] and for the *Voluntary Selection* condition [CHSX: M = 2.668; Control: M = 2.129; T = -1.144; p (one sided) = 0.131].

### Correlations

Correlations were calculated between different parameters (ERPs, behavioral data, demographical data, questionnaire results, substance use).

The reaction times (RTs) correlated significantly with the P3-amplitudes at Fz [*r* = 0.470; *p* = 0.010] and Cz [*r* = 0.474; *p* = 0.009] during *Voluntary Selection +*. All other correlations between ERPs (N1, N2, P3) and RTs during *Go* as well as *Voluntary Selection +* were not significant. There were no significant correlations between the ERPs and the demographical data, questionnaire results and substance use.

The reaction times during the *Go* condition and the *Voluntary Selection* task, and Chemsex related parameters, such as the number of years since the first Chemsex session [*Go*: *r* = -0.03; *p* = 0.278; *Voluntary Selection +*: *r* = -0.042; *p* = 0.883], the number of days since the last Chemsex session [*Go*: *r* = -0.072; *p* = 0.799; *Voluntary Selection +*: *r* = − 0.072; *p* = 0.799] did not correlate significantly. In addition, the behavioral data did not correlate with affective symptoms (MADRS, BDI) or hypersexuality (HBI) (Table 4). Correlations between reaction times and the frequency of substance use within the last six months for each substance (alcohol, nicotine, cannabis, amphetamine, methamphetamine, cocaine, GHB/GBL, ketamine, poppers) were not significant [all *p* > 0.05]. In the control group, RTs were associated with the number of days of alcohol consumption [r(*Go*) = 0.790; *p* = 0.011; r(*Voluntary selection +*) = 0.638; *p* = 0.064].

**Table 4:** Correlations between RTs and MADRS, BDI and HBI

	Chemsex group		Control group	
	r	*p*	r	*p*
RT (ms); MADRS				
Go	0.020	0.945	-0.471	0.089
Selection +	0.0201	0.473	-0.334	0.243
RT (ms); BDI				
Go	0.156	0.579	-0.544	0.044
Selection +	0.062	0.826	-0.702	0.005
RT (ms); HBI				
Go	-0.102	0.717	-0.434	0.121
Selection +	0.246	0.377	-0.357	0.211

RT = reaction time, ms = milliseconds, r = Pearson ratio = correlation coefficient, *p* = level of significance

## Discussion

The study is the first of its kind approaching Chemsex from a neurobiological perspective. Our research focused on comparing voluntary selection processes, inhibition, and neural correlates between Chemsex patients and a control group, using event-related potentials (ERPs) and behavioral data. To put the results in a clinical context, we collected data regarding demographics, substance use, sexuality, and depression/well-being.

Chemsex users showed increased scores on affective symptoms (BDI, MADRS) and reduced psychological well-being. These findings are consistent with previous studies that have reported decreased mental health and increased prevalence of depressions among Chemsex users [7, 53]. Nevertheless, these descriptive trends were not significant. Unexpectedly, no difference was observed in the self-rated physical well-being between the two study groups. In our study sexual risk behavior and hypersexuality were increased among the Chemsex group, indicating a potential association between Chemsex practices and heightened sexual impulsivity. In addition, a higher percentage of Chemsex users reported STDs including HIV, likely resulting from sexual high risk behavior [12]. These findings emphasize the necessity for targeted interventions, educational initiatives and harm-reduction strategies to address the potential health risks associated with Chemsex [54].

The participants rated the sensation of disinhibition experienced during a Chemsex session as very high. This emphasizes the primary motives of engaging in Chemsex, such as the enhancement or intensification of sexual experiences [4]. Regarding substances and addiction history, the Chemsex group primarily used substances in accordance with the defined drug criteria of Chemsex: methamphetamine, GHB/GBL, mephedrone, and ketamine. Notably, these findings are due to the fact that the usage of one of the four substances was a criterion for inclusion in the study. Still, out of all substances, apart from alcohol and nicotine, those four were the most prevalent among the Chemsex group. Additionally, cocaine and poppers were found to be more prevalent than other substances among Chemsex users.

Our clinical observations, particularly regarding mental health, underline the importance of comprehending the phenomenon of Chemsex, including its etiology and associated consequences, to provide effective assistance to affected individuals. The fact that 60% of the Chemsex users in our study expressed their desire to refrain from engagement in Chemsex, highlights the demand for therapeutic interventions and support mechanisms. Other studies [5] claim the lack of sexual protection and information as well.

Consistent with prior research [55, 56], our findings indicate that manual responses in the *Voluntary Selection +* are significantly slower compared to the *Go* condition. The act of free decision-making demands increased time consumption and a higher working memory load than automated response processes, as observed in the *Go* condition [57]. Chemsex users showed slightly longer response times after *Go* and *Voluntary Selection +* tasks in comparison to the control group. However, the difference was not significant, as was the difference in wrong responses. A similar pattern was found in a study on ADHD: the patients barely showed any differences to a control group regarding response time and number of mistakes [56]. After the *Voluntary Selection* stimulus Chemsex users responded by button press (*Selection +*) in 62.0% of trials, while the control group responded in 60.3% of trials, with no significant difference. One potential explanation is the exclusion of several participants (14 out of 43 subjects) who displayed inadequate comprehension of the task requirements. By including only individuals who exhibited good task performance, the outcomes may have been impacted. Another limitation of the study was the prevalence of subjects reporting difficulties in perceiving the auditory stimuli due to their low volume, which could have potentially affected participants’ performance.

The N2 amplitude has been linked to top-down inhibition of false responses [36] and response restraint [37, 39]. It is associated with stimulus classification in tasks involving free choice [58], high-conflict response trials [38], and response selection [59]. Accordingly, in our experiment, the N2 amplitudes were higher in the *NoGo* and *Voluntary Selection* conditions compared to the *Go* condition. They showed maximum prominence over the Fz and Cz electrodes, underlining the generation from the ACC [59]. Chemsex users showed reduced electrophysiological N2 responses, particularly in central regions. While those group differences were significant in decision-making tasks, they reached statistical trend level in behavioral inhibition. These results suggest dysfunction in frontocentral brain areas, which is consistent with prior findings in patients with substance use and impulsive disorders [21, 30]. These neurological alterations may contribute to risk behavior regarding drug abuse and explain differences concerning hypersexuality.

P3 amplitudes involve stimulus evaluation, categorization, motor response planning and serve as a link between stimulation and reaction [60]. The parietal P3 has been suggested to reflect attention towards *Go* stimuli, while the frontocentral P3 has been associated with inhibition during *NoGo* tasks to prevent premature responses [34, 37]. In our study, we found that the P3 component following *Go* stimuli was most prominent at Pz, whereas after *NoGo* stimuli, it was highest at Cz. There were no significant differences between the two groups in terms of parietal P3 (Pz) during the *Go* condition. The frontal P3 (Fz) showed slightly higher amplitudes in the control group during the *NoGo* condition. Overall, our findings suggest that the neurobiological mechanisms underlying the P3 component are not significantly influenced by Chemsex practices.

In this study, we gained valuable insights into the clinical and neurobiological effects of Chemsex. However, the causality remains uncertain, as it is unclear whether Chemsex leads to neurological alterations or if pre-existing neurological characteristics contribute to an inclination towards Chemsex practices. Some of the observed results may lack statistical significance due to intermittent substance abstinence among some of the Chemsex patients, since prior research indicates the potential reversibility of brain impairments [22, 25]. It is very common that so-called high consumption phases of Chemsex users are episodically replaced by abstinent phases [5]. To enhance the robustness of our findings, a follow-up study with a larger population size should be considered. This would allow for the replication of significant results and further exploration of observed tendencies. In addition, to minimize potential confounding effects, it would be advisable to match both groups for HIV status, as HIV can contribute to neurocognitive deficits and executive dysfunctions [61, 62]. Future studies should also incorporate a psychiatrically healthy control group to provide a more comprehensive comparison. In our study, an equal number of individuals with a psychiatric or psychotherapeutic history were presented in each group, which could induct bias. Some of the Chemsex patients were taking psychopharmaceuticals for treatment, such as Citalopram, which can affect behavioural responses by modulating attention [63]. The differences between the handedness of the participants (three left-handed Chemsex patients and one left-handed control subject) could have led to distortions in the results as well [64]. Nevertheless, we decided to leave all 29 subjects in the study because we did not want to further reduce the already small sample size. It is necessary to keep those factors in mind if a larger follow-up study should be implemented.

Understanding the neurophysiological alterations and identifying the affected brain regions, can offer valuable insights for developing targeted interventions. One promising approach is the application of non-invasive brain modulation interventions, such as transcranial direct current stimulation (tDCS), transcranial magnetic stimulation (rTMS), deep brain stimulation (DBS) and neurofeedback via EEG or functional magnetic resonance imaging (fMRI) [65, 66]. Studies investigating repeated tDCS of the dorsolateral prefrontal cortex (DLPFC) have demonstrated positive outcomes in individuals with alcohol, nicotine, and cocaine addiction: tDCS led to improvements in quality of life and reduction of consumption, craving, and anxiety [67]. Malandain et al. (2020) conducted the first case study exploring tDCS in severe Chemsex addiction with a positive short- and long-term outcome for the examined subject. Consequently, some of these therapeutic methods have shown promise in addiction treatment and may have the potential to reduce craving and minimize the negative outcomes associated with Chemsex. Still, further research is needed to investigate their benefits in addressing Chemsex addiction and associated neurobiological alterations [65, 67, 68].

## Conclusion

Difficulties in decision-making and inhibition of responses in Chemsex users seem to be associated with fronto-central deficits, while parietal brain functions seem to be less affected. Chemsex users exhibit trendwise increased scores on measures of depressive symptomatology and significantly higher rates in hypersexuality, along with reduced psychological well-being and increased sexual risk behavior. Comprehensive understanding of these clinical symptoms and the underlying neurophysiological processes can provide valuable perspectives on developing targeted interventions to minimize the negative consequences associated with Chemsex practices and improve overall well-being of Chemsex users.

## Electronic supplementary material

Below is the link to the electronic supplementary material.

Supplementary Material 1

Supplementary Material 2
