# Supplementary material for: What Chemsex does to the brain - neural correlates (ERP) regarding decision making, impulsivity and hypersexuality
Source: Eur Arch Psychiatry Clin Neurosci. 2024 Jul 6;275(1):227–40. doi: 10.1007/s00406-024-01856-2 (PMC11799082; doi:10.1007/s00406-024-01856-2)
Supplement: Supplementary file 2 — Supplementary Material 2 [file 406_2024_1856_MOESM2_ESM.docx]

**What Chemsex does to the brain - Neural correlates (ERP) regarding decision making, impulsivity and hypersexuality**

Johanna Schwarz^1,*,•^, Marcus Gertzen^2,•^, Andrea rabenstein^1^, Moritz Straßburger^1^, Alana Horstmann^1^, Oliver Pogarell^1^, Tobias Rüther^1,×^, Susanne Karch^1,×^

^1^ Department of Psychiatry and Psychotherapy, LMU University Hospital, LMU Munich, Germany

^2^ Department of Psychiatry, Psychotherapy, and Psychosomatics, Medical Faculty, University of Augsburg, Augsburg, Germany

^*^ Corresponding Author; J.Schwarz@med.uni-muenchen.de ^•^ Shared First Authors ^×^ Shared Last Authors

**Abstract**

Chemsex describes the use of specific substances (methamphetamine, GHB/GBL, mephedrone, ketamine) which initiate or enhance sexual experiences and is mainly prevalent among men who have sex with men. Many Chemsex users experience somatic complications (for example sexually transmitted diseases) and sometimes adverse sociological, psychological, and neurological symptoms, such as depression, impulse control disorders or hypersexuality. Changes in impulsivity and deficits in executive functions have demonstrated to be associated with addiction and impulse control disorders as well as frontal brain dysfunction and behavioral control deficits. This study aims to explore the effects of neurophysiological correlates of inhibition and decision making in Chemsex users with an EEG paradigm using event-related potentials (N2, P3). 15 Chemsex users and 14 matched control subjects, all of them men who have sex with man, participated in an auditory Go/NoGo/Voluntary Selection EEG paradigm. In addition, clinical data (e.g. regarding depression), demographic information as well as measures of well-being and sexual behavior were collected. The results demonstrated that clinical symptoms, hypersexuality, and sexual risk behaviour were more pronounced in Chemsex users compared to non-users. P3 amplitudes did not differ significantly between groups. However, the Chemsex users showed decreased electrophysiological N2 responses in fronto-central brain regions during decision-making, indicating compromised executive function and inhibitory control. The observed impairments may lead to increased risk behavior regarding drug abuse and hypersexuality. Understanding the neurobiological mechanisms can contribute to targeted interventions in order to mitigate the negative consequences of engaging in Chemsex and improve general well-being.

**Keywords**
Chemsex - Executive functions - Inhibition - Hypersexuality - Neurophysiological correlates

**Statements and Declarations**

This study is part of the Dr. med. thesis of Johanna Schwarz at the Faculty of Medicine, Ludwig-Maximilians-Universität, Munich. On behalf of all authors, the corresponding author Johanna Schwarz states that there is no conflict of interest. Also, the corresponding author states that the study has been approved by the ethics committee of LMU University Hospital and has been performed in accordance with the ethical standards laid down in the 1964 Declaration of Helsinki and its later amendments.

**Author Contributions**

All authors contributed to the study conception and design. Material preparation, data collection and analysis were mainly performed by Johanna Schwarz, Marcus Gertzen, Susanne Karch and Tobias Rüther. The first draft of the manuscript was written by Johanna Schwarz and all authors commented on previous versions of the manuscript. All authors read and approved the final manuscript. Johanna Schwarz and Marcus Gertzen contributed equally as shared first authors, Susanne Karch and Tobias Rüther contributed equally as shared last authors, Andrea Rabenstein contributed as co-author.
Conceptualization & Methodology: Johanna Schwarz, Marcus Gertzen, Susanne Karch, Tobias Rüther and Moritz Straßburger; Data collection: Johanna Schwarz and Moritz Straßburger; Formal analysis and investigation: Johanna Schwarz and Susanne Karch; Writing - original draft preparation: Johanna Schwarz; Writing - review and editing: Marcus Gertzen, Susanne Karch, Tobias Rüther, Andrea Rabenstein, Moritz Straßburger, Alana Horstmann, Oliver Pogarell; Supervision: Oliver Pogarell

Anonymous Data will be made available at reasonable request.

**Data deposition**

Anonymous Data will be made available at reasonable request.
