# Supplementary material for: What Chemsex does to the brain - neural correlates (ERP) regarding decision making, impulsivity and hypersexuality
Source: Eur Arch Psychiatry Clin Neurosci. 2024 Jul 6;275(1):227–40. doi: 10.1007/s00406-024-01856-2 (PMC11799082; doi:10.1007/s00406-024-01856-2)
Supplement: Supplementary file 1 — Supplementary Material 1 [file 406_2024_1856_MOESM1_ESM.docx]

| Klinik und Poliklinik für Psychiatrie und Psychotherapie  Nußbaumstraße 7, 80336 München  Germany |  |  |  | **Chairman**  Prof. Dr. med. Peter Falkai  **Contact Information**  Johanna Schwarz    Tel. +49 89 4400-55598  Mobile: +49 176 61739975  J.Schwarz@med.uni-muenchen.de  www.lmu-klinikum.de |
| --- | --- | --- | --- | --- |
| European Archives of Psychiatry and Clinical Neuroscience  Prof. Dr. med. Peter Falkai  Ludwig-Maximilians-University Munich Clinic for Psychiatry and Psychotherapy Nußbaumstr. 7 D-80336 Munich |  |  |  |  |
|  | | **06.01.2024** |  |  |

Dear Prof. Dr. Falkai,

Dear members of the editorial board,

On behalf of all authors of this manuscript, I wish to submit our original research article, "What Chemsex does to the brain - Neural correlates (ERP) regarding decision making, impulsivity and hypersexuality" for consideration and review by the European Archives of Psychiatry and Clinical Neuroscience.

The article presents the results of our clinical study examining the neural correlates of Chemsex as well as psychiatric consequences that result from this form of addiction. Not only is Chemsex a phenomenon that has been little studied clinically to date, but our study is the first to approach this topic from a neurobiological perspective. The understanding of the underlying neurophysiological processes and the clinical consequences can provide valuable perspectives on developing targeted interventions for Chemsex-users. We believe that this manuscript is appropriate for publication by your journal because it addresses an innovative and globally important topic, creating an interface between clinical psychiatry and cognitive neuroscience.

The ethics committee of LMU University Hospital approved this study, and a strict privacy protocol was applied to ensure participants’ anonymity. The study was conducted in accordance with the 1964 Declaration of Helsinki and its later amendments.

We confirm that this paper is an original work. It has not been published, nor is it currently under consideration for publication elsewhere.

All authors declare that they have no conflicts of interest relevant to this article.

Please don’t hesitate to address all correspondence regarding this manuscript to me at [J.Schwarz@med.uni-muenchen.de](mailto:J.Schwarz@med.uni-muenchen.de).

We thank you for taking this manuscript into your consideration.

Sincerely,

Johanna Schwarz

Junior Researcher, Department of Psychiatry and Psychotherapy

LMU University Hospital
